# The significance of blood indicators in distinguishing bipolar disorder depressive episodes from major depression in teenagers

**DOI:** 10.3389/fpsyt.2026.1865872

**Published:** 2026-08-06

**Authors:** Fangming Xu, Tingting Xie, Yu Su, Xia Feng

**Affiliations:** 1 Department of Sleep Medicine, the Second People’s Hospital of Guizhou Province, Guiyang, Guizhou, China; 2 Department of Psychiatry, the First Hospital of Hebei Medical University, Shijiazhuang, Hebei, China

**Keywords:** bipolar disorder depressive, complete blood count, derived inflammatory indicators, major depression disorder, nomogram prediction model, propensity score matching

## Abstract

**Purpose:**

There is ongoing debate about the role of blood indicators in distinguishing between bipolar disorder depressive episodes (BD-D) and major depressive disorder (MDD) in teenagers. This study aimed to assess the role of blood indicator in distinguishing BD-D from MDD in teenagers and identify potential biomarkers.

**Methods:**

We collected clinical data of BD-D and MDD teenagers from January 2022 to January 2025. To mitigate the effects of possible confounding variables, we performed the propensity score matching (PSM) analysis. After conducting univariate analysis, we performed logistic regression analysis, and ROC curve analysis on blood indicators, then nomogram prediction model was constructed using R language software.

**Results:**

A total of 275 patients were included in each group after PSM analysis. While many blood indicator were associated with by univariate analysis, logistic regression analysis identified the lymphocyte-to-HDL ratio (LHR, p<0.0001) and platelet-to-HDL ratio (PHR, p<0.0001) as predictors for BD-D. ROC curve analysis further revealed 1.3307 and 108.3806 as the critical values for separating BD-D from MDD in teenagers. Subsequently, we established nomogram-based prediction model and obtained an AUC of 0.7097 with the calibration curve showing strong consistency and decision curve analysis indicating substantial clinical utility.

**Conclusions:**

Our investigation revealed that LHR and PHR levels were correlated with BD-D and could be considered as potential biomarkers, which was confirmed by the nomogram prediction model. We also determined their threshold values to distinguish BD-D from MDD in teenagers. We should recognize that these results still necessitate further clinical validation.

## Introduction

Approximately 2-3% of the population are affected by bipolar disorder (BD), while major depressive disorder (MDD) impacts up to 10%, both being chronic and recurrent mood disorders (1, 2). BD, a crucial mental health issue, predominantly arises during adolescence and frequently involves high impulsivity, hypersexuality, and risky sexual conduct (3–5). MDD is characterized solely by depressive and dysthymic episodes (1, 2). Around 60% of people with BD show symptoms of a major depressive episode before their first hypo/manic episode (6, 7). Therefore, BD is commonly diagnosed late and is confused with MDD in about 40% of instances (8). Misdiagnosed BD patients might use antidepressants, which may heighten the risk of mania and postpone the accurate diagnosis (9, 10).

Increasing evidence from recent studies suggests that immune dysregulation is linked to mood disorders (11), emphasizing the role of the inflammatory response system activation as a key pathological factor (12). Current research has increasingly focused on showing that tumor necrosis factor (TNF)-α and interleukin (IL)-6 levels are associated with MDD (13). Other investigations have discovered connections between IL-6, TNF-α, and higher ratios of TNF-α/IL-4, IL-2/IL-4, and interferon (IFN)-γ/IL-4, which were associated with BD (14).Current studies, to the best of our knowledge, concentrate on the immunological differences between adult BD-D and MDD, while adolescents are not given much consideration. Consequently, this study seeks to observe the disparities in blood indicators among adolescent BD-D and MDD patients to explore BD-D biomarkers.

## Materials and methods

### Ethics statement

Before data collection, this study was approved by the institutional review boards of First Hospital of Hebei Medical University (20220908) in compliance with the Helsinki and an exemption from the informed consent was obtained. All data were anonymized before the analysis to safeguard patient privacy.

From January 2022 to January 2025, we collected retrospective data on teenagers with BD-D and MDD, categorizing them into the BD-D and MDD groups, respectively. The collection of blood samples for the two groups occurred within the time window of 6:00 to 8:00 a.m. The retrospective nature of the study meant that formal structured diagnostic interviews such as the MINI-Kid were not utilized; however, all patients were evaluated using a standardized clinical approach based on the diagnostic criteria of the 10th Revision of the International Statistical Classification of Diseases and Related Health Problems (ICD-10), including psychiatric evaluations, review of healthcare records, and input from parents or guardians. The study involved diagnoses being confirmed by two psychiatrists who each had over five years of experience in child and adolescent psychiatry. When they disagreed, a third senior psychiatrist was consulted to reach a consensus.

The inclusion criteria specified: (1) individuals who are 13 to 19 years old; (2) diagnosed with BD-D and MDD according to ICD-10; (3) teenagers visiting the hospital for the first time, without receiving psychotropic medications (antidepressants, mood stabilizers, antipsychotics) or other drugs that may affect inflammatory markers. The criteria for exclusion comprised: (1) individuals suffering from other mental disorders, such as schizophrenia; (2) those with conditions that affect inflammatory markers, including acute inflammatory and metabolic diseases; (3) to reduce the risk of misclassification, patients with unusual symptoms or uncertain diagnostic criteria were not included in the study; (4) incomplete data.

The demographic data included age, gender, body mass index (BMI), disease duration, as well as the a history of family, smoking, and alcohol. Admission laboratory indicators covered white blood cell (WBC, 10^9^/L), neutrophil (NEU, 10^9^/L), lymphocyte (LYM, 10^9^/L), monocyte (MON, 10^9^/L), platelet (PLT, 10^9^/L), high-density lipoprotein (HDL, mmol/L); C-reactive protein (CRP, mg/L), interleukin-6 (IL6, pg/L), erythrocyte sedimentation rate (ESR, mm/h); PCT (procalcitonin, ng/L), neutrophil-tolymphocyte ratio (NLR), monocyte-to-lymphocyte ratio (MLR), platelet-to-lymphocyte ratio (PLR), neutrophil-to-HDL ratio (NHR), lymphocyte-to-HDL ratio (LHR), monocyte-to-HDL ratio (MHR), platelet-to-HDL ratio (PHR), systemic immuneinflammation index (SII, SII=platelets count×neutrophils count/lymphocytes count), and system inflammation response index (SIRI, SIRI=monocyte count×neutrophils count/lymphocytes count).

### Statistics

We utilized SPSS (version 27.0, SPSS Inc., Chicago, IL) and considered p<0.05 to indicate statistical significance. We applied the Mann-Whitney U test because the variables in our study did not adhere to normality criteria. The chi-square test was used for analyzing count data. To address differences in baseline characteristics and minimize selection bias, a 1:1 ratio propensity score matching (PSM) analysis was conducted between the groups with of caliper width of 0.1, matching variables (age, gender, body mass index, disease duration, and family history, as well as a history of smoking and alcohol), and balance diagnostics before and after matching (|SMD| < 0.1). Logistic regression analysis, incorporating patient characteristics as covariates, was used to determine PSM. After establishing PSM, we used univariate and logistic regression analyses to compare blood test results at admission between BD-D and MDD. Ultimately, the creation of the nomogram prediction model for BD-D involved the use of R language software, and the model’s discriminative power was evaluated using the AUC of the ROC, and we utilized a calibration curve to compare the forecasted and real probabilities of this prediction model. The decision curve analysis was performed to assess the model’s clinical utility.

## Results

**Figure 1:**
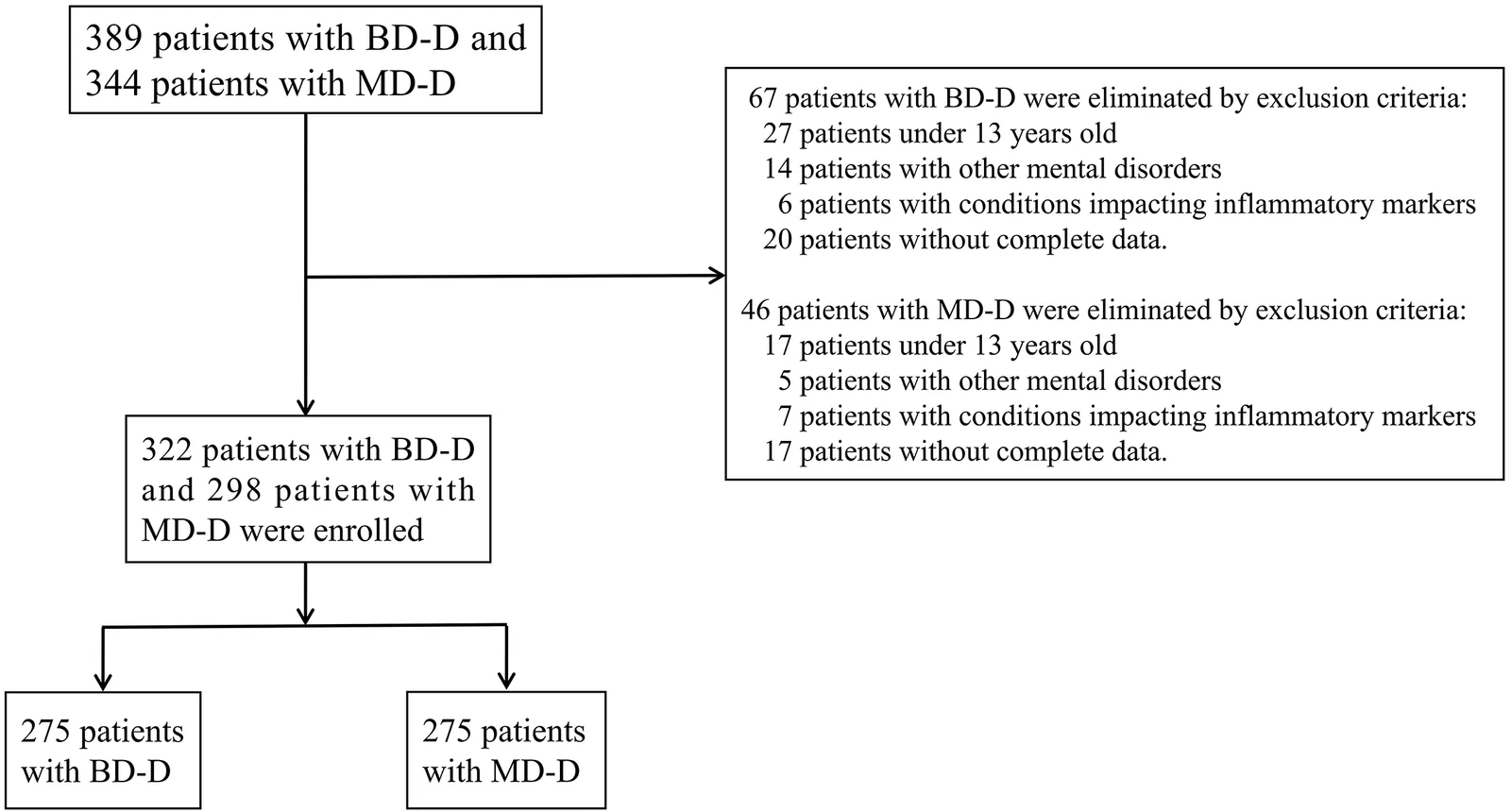
Flow diagram of included patients.

The study involved 389 BD-D patients and 344 MDD patients between January 2022 and January 2025. Following exclusion criteria, 67 BD-D and 46 MDD patients were removed (**Figure 1**). The study eventually comprised 322 patients diagnosed with BD-D and 298 with MDD. **Table 1** presents the baseline characteristics for each group. There were significant differences in age (p=0.046) and sex (p=0.049) prior to PSM, but the baseline characteristics were uniform after PSM (all p > 0.05).

**Table 1:** Patient characteristics at baseline.

Variables	Pre-matching			Post-matching		
	BD-D (n=322)	MDD (n=298)	*p*	BD-D (n=275)	MDD (n=275)	*p*
Age, years	15.0 (14.0~16.0)	15.5 (15.0~16.0)	0.046	15.0 (15.0~16.0)	16.0 (15.0~17.0)	0.076
Gender			0.049			0.291
male	101	116		98	110	
female	221	182		177	165	
Body mass index (kg/m2)	22.0(20.2~23.6)	22.1(20.2~23.8)	0.708	22.1(20.4~23.8)	22.1(20.3~24.0)	0.829
Disease duration	2.0 (2.0~2.0)	2.0 (2.0~2.0)	0.880	2.0 (2.0~2.0)	2.0 (2.0~2.0)	0.242
Family history			0.496			0.417
Yes	81	68		67	59	
No	241	230		208	216	
Smoking			0.877			0.600
Yes	44	42		35	31	
No	278	256		240	244	
Alcohol			0.806			0.382
Yes	41	36		23	29	
No	281	262		252	246	

BD-D, bipolar disorder depressive episodes; MDD, major depression disorder.

**Table 2** indicates that LYM, LHR, MHR, and PHR levels were notably higher in the BD-D group, while the MDD group had significantly increased levels of HDL, CRP, IL-6, PCT, NLR, PLR, MLR, SII, and SIRI (all p<0.05). Additionally, our logistic regression analysis indicated that the levels of LHR [p<0.0001, OR = 8.233, 95%CI (4.078, 16.619)] and PHR [p<0.0001, OR = 1.024, 95%CI (1.011, 1.036)] independently contributed to the risk of BD-D (**Table 3**). The ROC curve analysis demonstrated that the levels of LHR [p<0.0001, AUC area=0.694, 95%CI(0.650, 0.739)] and PHR [p<0.0001, AUC area=0.646, 95%CI(0.600, 0.692)] acted as independent associated factors for BD-D. The cut-off points for LHR and PHR to differentiate BD-D from MDD were identified as 1.3307 and 108.3806, respectively (**Table 4**).

**Table 2:** Comparison of laboratory variables between BD-D and MDD by univariate analysis.

Laboratory variables	BD-D (n=275)	MDD (n=275)	p
WBC (10^9^/L)	6.79(6.25~7.41)	6.65(6.14~7.35)	0.174
LYM (10^9^L)	2.22(1.90~2.68)	2.05(1.76~2.30)	<0.0001
MON (10^9^/L)	2.47(2.04~2.87)	2.50(2.07~2.91)	0.693
NEU (10^9^/L)	2.11(1.52~2.55)	2.19(1.42~2.94)	0.077
HDL (mmol/L)	1.95(1.77~2.06)	2.00(1.91~2.09)	<0.0001
PLT (10^9^/L)	181.0(166.0~196.0)	178.0(164.0~194.0)	0.132
CRP (mg/L)	5.5(4.9~6.2)	5.8(5.1~6.5)	0.002
IL-6 (pg/L)	9.5(8.5~10.6)	9.8(8.8~10.8)	0.025
PCT (ng/L)	0.701(0.698~0.701)	0.72(0.715~0.725)	<0.0001
ESR (mm/h)	43.0(40.0~46.0)	44.0(40.0~48.0)	0.106
NLR	0.86(0.59~1.26)	1.04(0.68~1.54)	<0.0001
PLR	80.4(64.5~99.4)	87.1(76.3~103.1)	<0.0001
MLR	1.06(0.82~1.41)	1.26(0.97~1.54)	<0.0001
NHR	1.14(0.87~1.46)	1.10(0.69~1.49)	0.203
LHR	1.16(0.99~1.59)	1.00(0.87~1.15)	<0.0001
MHR	1.34(1.09~1.63)	1.22(1.03~1.46)	<0.0001
PHR	95.6(85.9~111.8)	89.0(81.8~98.0)	<0.0001
SII	157.8(104.7~227.2)	196.1(111.1~278.1)	0.002
SIRI	2.02(1.43~3.08)	2.49(1.59~3.50)	0.001

BD-D, bipolar disorder depressive episodes; MDD, major depression disorder; WBC, white blood cell; NEU, neutrophil; LYM, lymphocyte; MON, monocyte; HDL, high-density lipoprotein; PLT, platelet; IL6, interleukin-6; CRP, C-reactive protein; PCT, procalcitonin; ESR, erythrocyte sedimentation rate; NLR, Neutrophil to Lymphocyte Ratio; MLR, Monocyte-to-lymphocyte ratio; PLR, Platelet-to-lymphocyte ratio; NHR, neutrophils count/HDL count; LHR, lymphocyte count/HDL count; MHR, monocytes count/HDL count; PHR, platelets count/HDL count; SII, (platelets count × neutrophils count)/lymphocytes count; SIRI, (monocyte count×neutrophils count)/lymphocytes count; SII, systemic immune-inflammation index; SIRI, system inflammation response index.

**Table 3:** Logistic regression analysis of laboratory variables between BD-D and MD-D.

Variables	OR	95%CI		P value
		Lower limit	Upper limit	
LHR	8.233	4.078	16.619	<0.0001
PHR	1.024	1.011	1.036	<0.0001

BD-D, bipolar disorder depressive episodes; MD-D, major depression disorder; LHR, lymphocyte-to-HDL ratio; PHR, Platelet-to-HDL ratio.

**Table 4:** ROC curve analysis and cut-off values.

Variables	Area	P-value	95%CI		Cut-off value
			Lower limit	Upper limit	
LHR	0.694	<0.0001	0.650	0.739	1.3307
PHR	0.646	<0.0001	0.600	0.692	108.3806

LHR, lymphocyte-to-HDL ratio; PHR, Platelet-to-HDL ratio.

**Figure 2:**
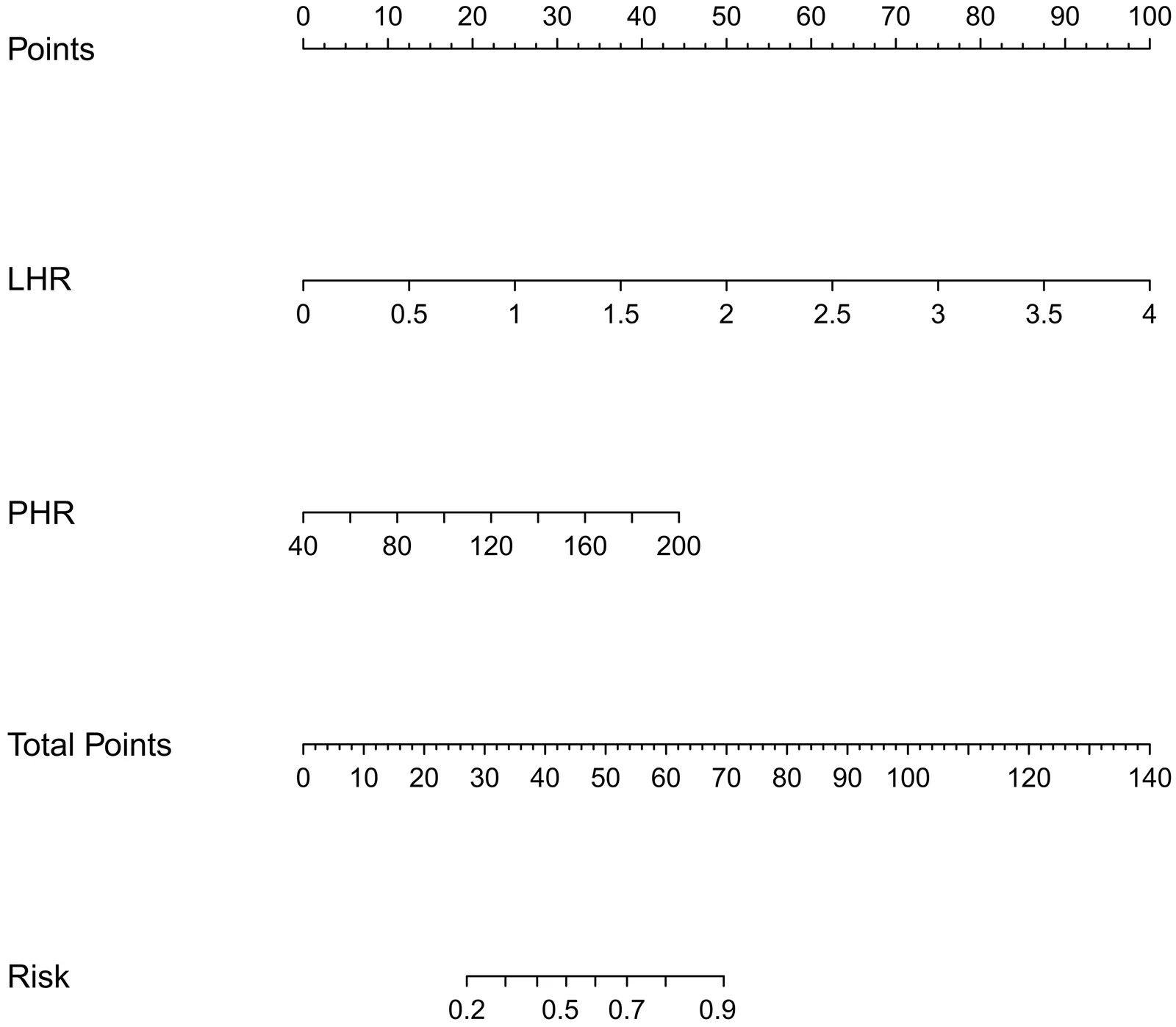
The nomogram prediction model for bipolar disorder depressive episodes.

**Figure 3:**
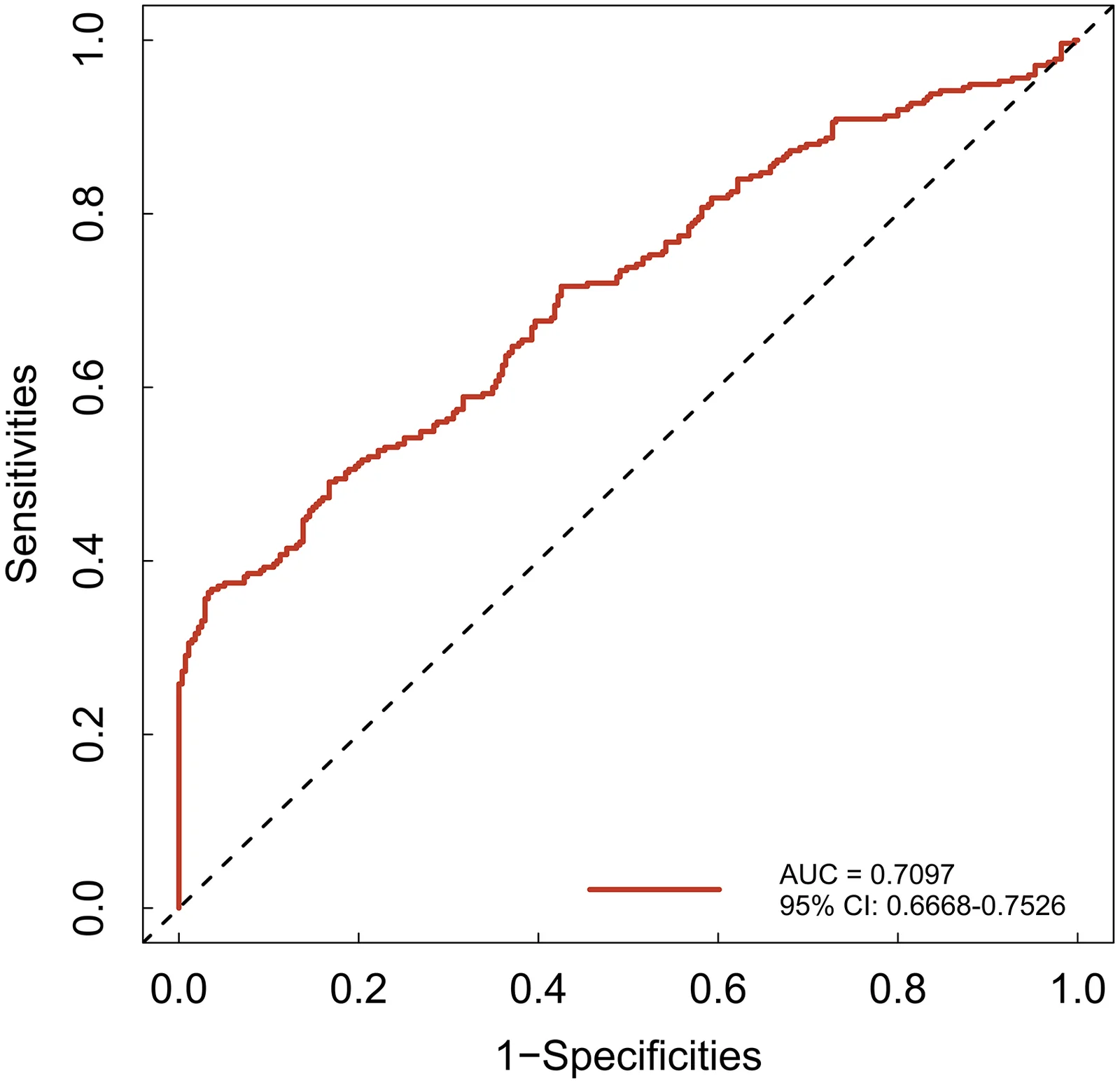
The receiver operating characteristic curves of the nomogram.

**Figure 4:**
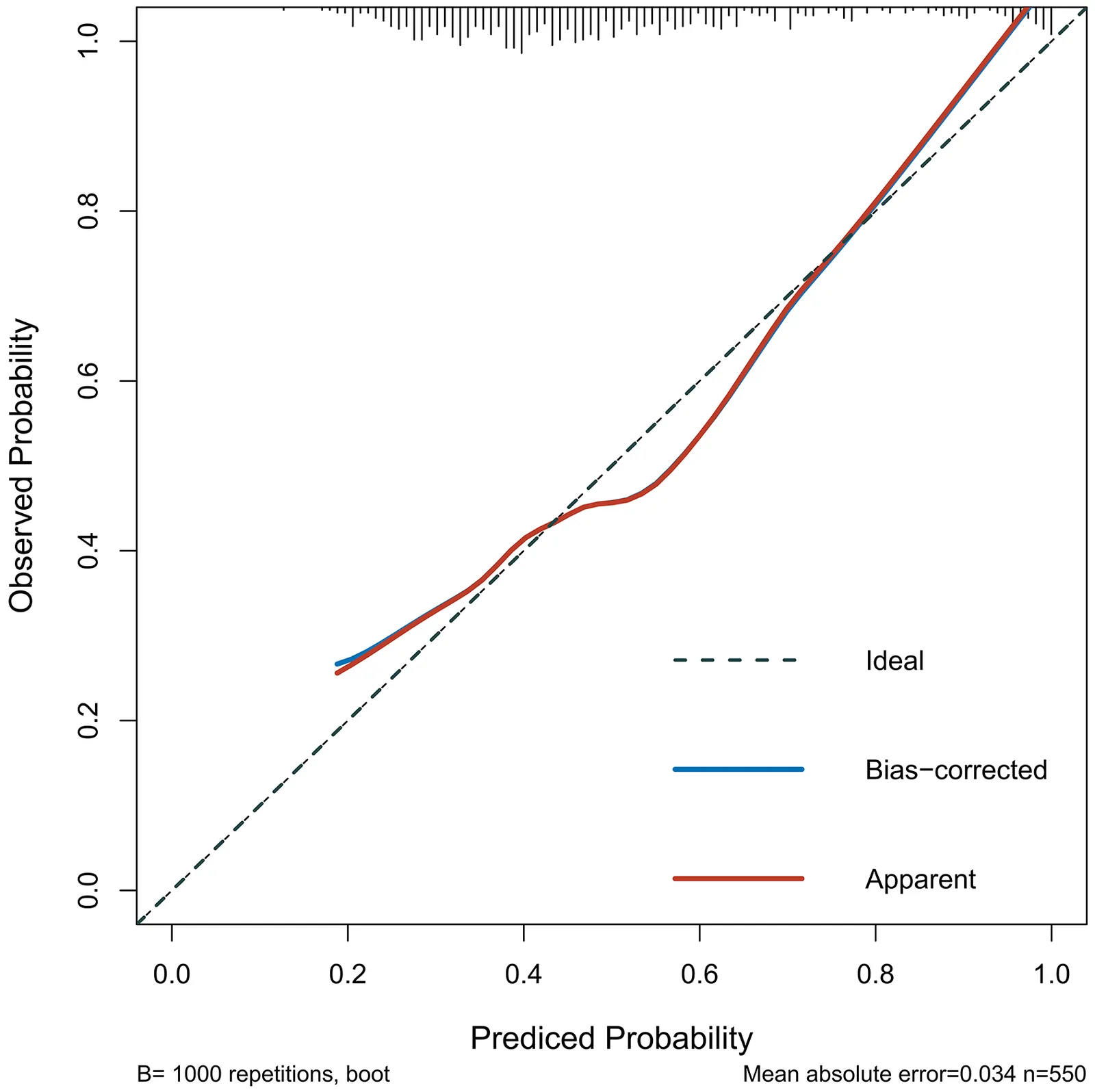
The calibration curve of the nomogram.

**Figure 5:**
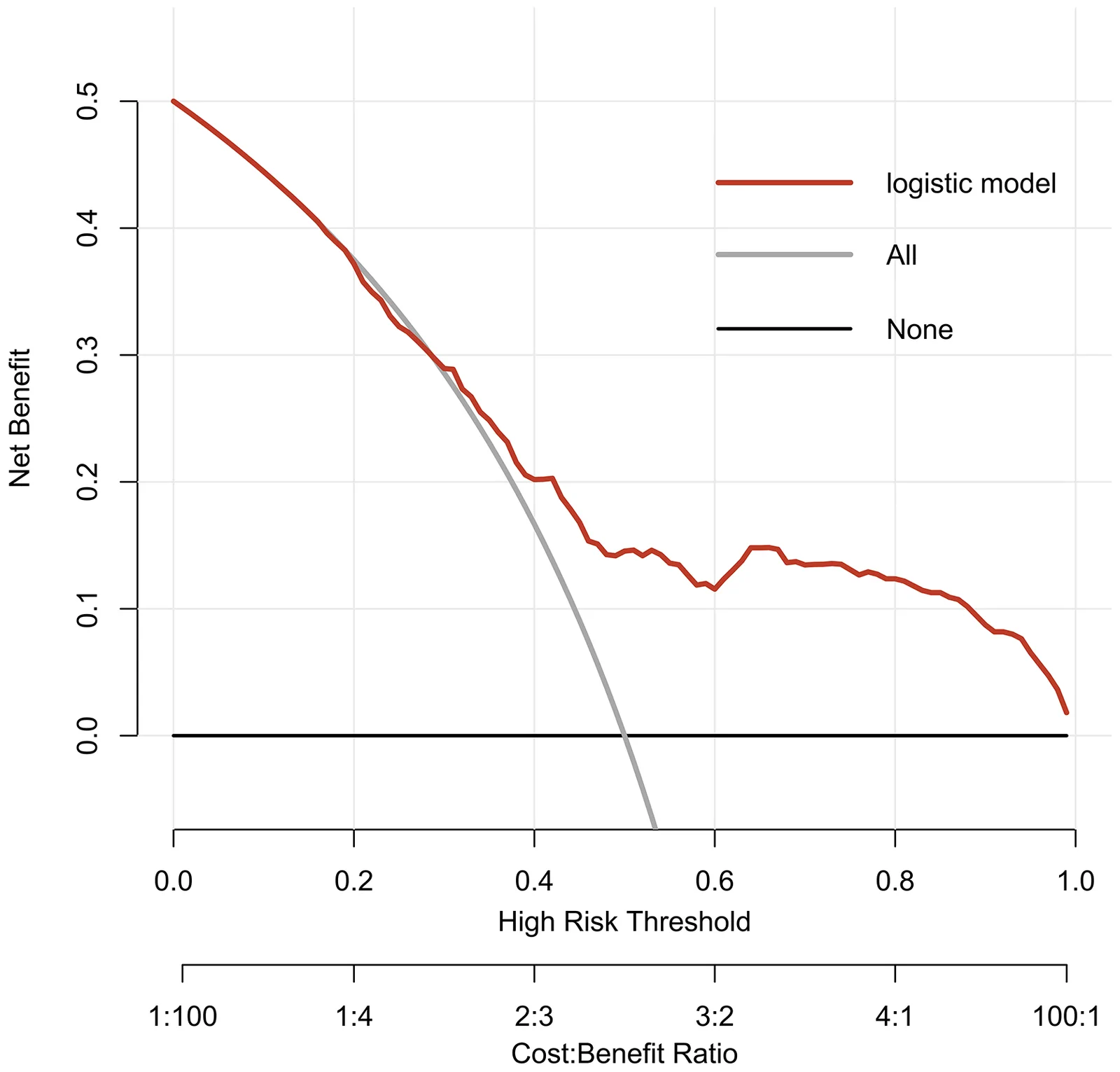
The decision curve analysis of the nomogram.

Based on **Figure 2**, we created a nomogram prediction model using logistic regression analysis. **Figures 3** and **4** indicate that the ROC curve of the nomogram demonstrates moderate discrimination capability [AUC = 0.7079, 95%CI(0.6668, 0.7526)]. According to the Hosmer-Lemeshow goodness of fit test, the nomogram’s calibration curve was accurately calibrated. As depicted in **Figure 5**, the decision curve analysis indicated that our nomogram prediction model offers moderate clinical benefits.

## Discussion

Clinicians find it hard to differentiate between BD-D and MDD diagnoses because approximately 60% of BD patients experience major depressive episode symptoms before their first hypo/manic episode (6, 7). The substantial difference in treatment for the two conditions means that a misdiagnosis might aggravate symptoms, result in serious repercussions for patients and their families, and heighten the economic strain on society. As a result, early identification of the two diseases holds great importance. Recent research has focused more on proving that TNF-α and IL-6 levels are linked to MDD (13), while other studies have shown that IL-6 and TNF-α are linked to BD (14). According to current understanding, the immunological differences between teenage BD-D and MDD have not been adequately addressed. Therefore, the study intends to investigate the variations in blood indicators among adolescent BD-D and MDD patients to discover BD-D biomarkers.

Our study revealed that while numerous laboratory indicators were associated with BD-D by univariate analysis, logistic regression analysis highlighted LHR and PHR levels as associated factors. According to the ROC curve analysis, 1.3307 and 108.3806 served as the cut-off points for predicting BD-D from MDD. Subsequently, we developed a nomogram prediction model with an AUC of 0.7079, demonstrating moderate consistency in the calibration curve and certain clinical applicability, as shown by decision curve analysis.

The production of pro-inflammatory and pro-oxidant cytokines by Mon is essential for the innate immune system’s reaction to pathogens (15). There is an association between affective disorders and increased monocyte counts along with alterations (16, 17). CRP is a pentameric acute-phase protein produced by the liver and enters the bloodstream. It plays a crucial role in the innate immune system, as its levels increasing quickly during infection and inflammation and decreasing rapidly after the acute phase. Fernandes (18) found that CRP levels were elevated in BD patients compared to healthy people, regardless of their mood states, with BD-M patients having the highest levels. IL-6 serves as a key cytokine involved in both immune system regulation and inflammatory responses. Studies have previously indicated that IL-6 levels are considerably higher in BD patients, although they do not rise significantly in BD-D subjects relative to healthy individuals (19).

Studies conducted recently have shown that lymphocyte subtypes, including Breg, Treg, and natural killer cells, are essential in the progression of affective disorders (20, 21). It has been proposed that these subtypes serve as markers of inflammation, which supports the inflammatory hypothesis concerning the development of these conditions. The study by Breunis and Barbosa found that individuals with symptomatic and remitted BD exhibited elevated levels of activated CD3+ T cells compared to those without the disorder (16, 22). The research conducted by Wu showed a substantial difference in T cell distribution between BD and MDD patients, with BD patients displaying significantly reduced levels of cytotoxic T cells compared to MDD patients (21, 23). Chourpiliadis (24) conducted a longitudinal cohort study on a population basis and discovered that higher glucose levels and reduced HDL-C levels were linked to a greater risk of common psychiatric disorders such as depression, or stress-related disorders. PCT acts as a precursor to the hormone calcitonin and is secreted by a variety of parenchymal cells, representing the body’s overall inflammatory response (25). This study revealed that LYM levels were significantly elevated in the BD-D group, whereas the MDD group showed a marked increase in HDL, CRP, IL-6, and PCT levels according to univariate analysis. However, no significant differences were observed in these indicators.

NLR, PLR, MLR, NHR, LHR, MHR, PHR, SII, and SIRI are indices that are both cost-effective and easy to use, suggesting a balance of systemic inflammation in a range of diseases (26–29). Numerous studies are currently examining the connection between these indicators and mood disorders. The indices NLR, MLR, and SII were notably elevated in BD patients compared to healthy individuals, suggesting increased inflammation (30). Adolescents with MDD show a correlation with high NPR, especially among females and those who are overweight (31). Cavaleri (32) recently conducted a meta-analysis to evaluate the differences in NLR, MLR, and PLR between MDD and BD-D, identifying these indicators as potential predictors for distinguishing BD-D from MDD. However, these predictors were not the biomarkers in our study. Two possible explanations for this variation are: the age difference of the patients, with the article examining adults and our research focusing on adolescents, and racial differences. As far as we are aware, there are not many studies examining the differences in these inflammatory markers between adolescent BD-D and MDD. Our results demonstrated that LHR and PHR are predictors for BD-D from the MDD group, and we identified their cut-off values, suggesting their role in helping clinicians differentiate BD-D from MDD. By combining white blood cell count with HDL, LHR and PHR serve as composite indicators that offer a more thorough reflection of the body’s inflammatory and metabolic conditions. Although there is currently no direct evidence to suggest that LHR/PHR can directly cause bipolar disorder, abnormal inflammatory metabolic states (such as abnormally elevated PHR) may indirectly participate in the pathological and physiological processes of bipolar disorder by affecting mechanisms such as neuroinflammation and blood-brain barrier permeability.

Additionally, our goal was to determine the combined predictors that most accurately distinguish BD-D patients from MDD by utilizing inflammatory markers. According to our findings, LHR+PHR proved to be the most accurate in predicting BD-D from MDD in teenagers. Emphasizing their cut-off values can greatly assist in the early differentiation of BD-D patients. To further verify these findings, we still need a larger sample size. Subsequently, we created a prediction model to assess BD-D from MDD. The ROC curve indicated effective differentiation, and the calibration curve was accurately aligned. Decision curve analysis showed that our nomogram prediction model is highly relevant in clinical settings. We should consider the absence of both internal and external validation due to small sample size, which may affect the accuracy of our predictive model. Hence, more validation via multicenter and large-scale follow-up studies is essential.

While this study offers some groundbreaking insights, it is important to acknowledge certain limitations. The investigation was carried out at a single center with a small sample size, so we did not conduct internal and external validation. Thus, conducting a multicenter randomized controlled trial with an increased number of participant is essential. Furthermore, the constraints of data collection in a retrospective study impact certain significant inflammatory markers, such as medication exposure, treatment history, dietary habits, and other clinical or lifestyle variables. Furthermore, differences in symptom severity between groups could influence inflammatory markers. Third, Since some patients with MDD might eventually develop BD-D, our analysis was restricted to the initial diagnosis at admission. Fourth, single-time-point biomarker assessment, residual confounding, and lack of healthy controls should be considered.

In summary, PSM was employed to minimize the impact of possible confounders and investigate the significance of blood markers at admission. We discovered that LHR and PHR levels were associated with BD-D for BD-D, unlike MDD. Additionally, we established their cut-off values and formulated a nomogram prediction model to effectively predict BD-D from MDD. Nonetheless, we must consider that further clinical validation is still needed for the current results.

## Data Availability

The original contributions presented in the study are included in the article/supplementary material. Further inquiries can be directed to the corresponding author.

## References

[1] Grande I Berk M Birmaher B Vieta E . Bipolar disorder. Lancet. (2016) 387:1561–72. doi: 10.1016/s0140-6736(15)00241-x 26388529

[2] Kupfer DJ Frank E Phillips ML . Major depressive disorder: new clinical, neurobiological, and treatment perspectives. Lancet. (2012) 379:1045–55. doi: 10.1176/appi.focus.12.2.217 22189047 PMC3397431

[3] Perlis RH Miyahara S Marangell LB Wisniewski SR Ostacher M DelBello MP . Long-term implications of early onset in bipolar disorder: data from the first 1000 participants in the systematic treatment enhancement program for bipolar disorder (STEP-BD). Biol Psychiat. (2004) 55:875–81. doi: 10.1016/j.biopsych.2004.01.022 15110730

[4] Kopeykina I Kim HJ Khatun T Boland J Haeri S Cohen LJ . Hypersexuality and couple relationships in bipolar disorder: a review. J Afect Disord. (2016) 195:1–14. doi: 10.1016/j.jad.2016.01.035 26851616

[5] Dvorak RD Wray TB Kuvaas NJ Kilwein TM . Mania and sexual risk: associations with behavioral self-regulation. J Afect Disord. (2013) 150:1076–81. doi: 10.1016/j.jad.2013.04.023 23721925

[6] Dudek D Siwek M Zielińska D Jaeschke R Rybakowski J . Diagnostic conversions from major depressive disorder into bipolar disorder in an outpatient setting: results of a retrospective chart review. J Affect Disord. (2013) 144:112–5. doi: 10.1016/j.jad.2012.06.014 22871536

[7] Baldessarini RJ Tondo L Visioli C . First-episode types in bipolar disorder: predictive associations with later illness. Acta Psychiatr Scand. (2014) 129:383–92. doi: 10.1111/acps.12204 24152091

[8] Fritz K Russell AMT Allwang C Kuiper S Lampe L Malhi GS . Is a delay in the diagnosis of bipolar disorder inevitable? Bipolar Disord. (2017) 19:396–400. doi: 10.1111/bdi.12499 28544121

[9] Morselli PL Elgie R GAMIAN-Europe/BEAM survey I . Global analysis of a patient questionnaire circulated to 3450 members of 12 European advocacy groups operating in the field of mood disorders. Bipolar Disord. (2003) 5:265–78. doi: 10.1034/j.1399-5618.2003.00037.x 12895204

[10] Sidor MM Macqueen GM . Antidepressants for the acute treatment of bipolar depression: a systematic review and meta-analysis. J Clin Psychiatry. (2011) 72:156–67. doi: 10.4088/jcp.09r05385gre 21034686

[11] Blume J Douglas SD Evans DL . Immune suppression and immune activation in depression. Brain Behav Immun. (2011) 25:221–9. doi: 10.1016/j.bbi.2010.10.008 20955778 PMC3025086

[12] Simon MS Schiweck C Arteaga-Henríquez G Poletti S Haarman BCM Dik WA . Monocyte mitochondrial dysfunction, inflammaging, and inflammatory pyroptosis in major depression. Prog Neuro-Psychopharmacol Biol Psychiatry. (2021) 111:110391. doi: 10.1016/j.pnpbp.2021.110391 34171401

[13] Liu Y Ho RC Mak A . Interleukin (IL)-6, tumour necrosis factor alpha (TNF-α) and soluble interleukin-2 receptors (sIL-2R) are elevated in patients with major depressive disorder: a meta-analysis and meta-regression. J Affect Disord. (2012) 139:230–9. doi: 10.1016/j.jad.2011.08.003 21872339

[14] Kim YK Jung HG Myint AM Kim H Park SH . Imbalance between pro-inflammatory and anti-inflammatory cytokines in bipolar disorder. J Affect Disord. (2007) 104:91–5. doi: 10.1016/j.jad.2007.02.018 17434599

[15] Pfau ML Ménard C Russo SJ . Inffammatory mediators in mood disorders: therapeutic opportunities. Annu Rev Pharmacol Toxicol. (2018) 58:411–28. doi: 10.1146/annurev-pharmtox-010617-052823 28992428 PMC5826556

[16] Barbosa IG Rocha NP Assis F Vieira ÉL Soares JC Bauer ME . Monocyte and lymphocyte activation in bipolar disorder: a new piece in the puzzle of immune dysfunction in mood disorders. Int J Neuropsychopharmacol. (2014) 18:pyu021. doi: 10.1093/ijnp/pyu021 25539506 PMC4368866

[17] Hughes HK Ashwood P . Overlapping evidence of innate immune dysfunction in psychotic and affective disorders. Brain Behav Immun Health. (2020) 2:100038. doi: 10.1016/j.bbih.2020.100038 34589829 PMC8474635

[18] Fernandes BS Steiner J Molendijk ML Dodd S Nardin P Gonçalves CA . C-reactive protein concentrations across the mood spectrum in bipolar disorder: a systematic review and meta-analysis. Lancet Psychiatry. (2016) 3:1147–56. doi: 10.1016/s2215-0366(16)30370-4 27838212

[19] Xu F Su Y Wang X Zhang T Xie T Wang Y . Olink proteomics analysis uncovers inflammatory proteins in patients with different states of bipolar disorder. Int Immunopharmacol. (2024) 131:111816. doi: 10.1016/j.intimp.2024.111816 38484669

[20] Maes M . Depression is an inflammatory disease, but cell-mediated immune activation is the key component of depression. Prog Neuro-Psychopharmacol Biol Psychiatry. (2011) 35:664–75. doi: 10.1016/j.pnpbp.2010.06.014 20599581

[21] Becking K Haarman BCM Grosse L Nolen WA Claes S Arolt V . The circulating levels of CD4+ t helper cells are higher in bipolar disorder as compared to major depressive disorder. J Neuroimmunol. (2018) 319:28–36. doi: 10.1016/j.jneuroim.2018.03.004 29685287

[22] Breunis MN Kupka RW Nolen WA Suppes T Denicoff KD Leverich GS . High numbers of circulating activated T cells and raised levels of serum IL-2 receptor in bipolar disorder. Biol Psychiatry. (2003) 53:157–65. doi: 10.1016/s0006-3223(02)01452-x 12547472

[23] Wu W Zheng YL Tian LP Lai JB Hu CC Zhang P . Circulating T lymphocyte subsets, cytokines, and immune checkpoint inhibitors in patients with bipolar II or major depression: a preliminary study. Sci Rep. (2017) 7:1–7. doi: 10.1038/srep40530 28074937 PMC5225421

[24] Chourpiliadis C Zeng Y Lovik A Wei D Valdimarsdóttir U Song H . Metabolic profile and long-term risk of depression, anxiety, and stress-related disorders. JAMA Netw Open. (2024) 7:e244525. doi: 10.1001/jamanetworkopen.2024.4525 38564219 PMC10988352

[25] Assicot M Gendrel D Carsin H Raymond J Guilbaud J Bohuon C . High serum procalcitonin concentrations in patients with sepsis and infection. Lancet. (1993) 341:515–8. doi: 10.1016/0140-6736(93)90277-n 8094770 PMC7141580

[26] Bittoni A Pecci F Mentrasti G Crocetti S Lupi A Lanese A . Systemic immune-inflammation index: a prognostic tiebreaker among all in advanced pancreatic cancer. Ann Transl Med. (2021) 9:251. doi: 10.21037/atm-20-3499 33708878 PMC7940927

[27] Wang J Zhou D Dai Z Li X . Association between systemic immune-inflammation index and diabetic depression. Clin Interv Aging. (2021) 16:97–105. doi: 10.2147/cia.s285000 33469277 PMC7810592

[28] Zhou YX Li WC Xia SH Xiang T Tang C Luo JL . Predictive value of the systemic immune inflammation index for adverse outcomes in patients with acute ischemic stroke. Front Neurol. (2022) 13:836595. doi: 10.3389/fneur.2022.836595 35370926 PMC8971364

[29] Cakir N Koc AN . Gamma-glutamyl transpeptidase-platelet ratio, systemic immune inflammation index, and system inflammation response index in invasive aspergillosis. Rev Assoc Med Bras. (2021) 67:1021–5. doi: 10.1590/1806-9282.20210475 34817517

[30] Dadouli K Janho MB Hatziefthimiou A Voulgaridi I Piaha K Anagnostopoulos L . Neutrophil-to-lymphocyte, monocyte-to-lymphocyte, platelet-to-lymphocyte ratio and systemic immune-inflammatory index in different states of bipolar disorder. Brain Sci. (2022) 12:1034. doi: 10.3390/brainsci12081034 36009097 PMC9405738

[31] Geng D Wang W Du N Niwenahisemo LC Xu H Wang Y . Association of the neutrophil-to-platelet ratio with response to electroconvulsive therapy in adolescents with major depressive disorder. Front Psychiatry. (2024) 15:1413608. doi: 10.3389/fpsyt.2024.1413608 39655209 PMC11625731

[32] Cavaleri D Cucchi G Citton M Monti M Crocamo C Bartoli F . Complete blood count-based inflammatory ratios in people with bipolar disorder: a systematic review and meta-analysis of neutrophil-to-lymphocyte, monocyte-to-lymphocyte, and platelet-to-lymphocyte ratios. Eur Psychiatry. (2026) 69:e26. doi: 10.1192/j.eurpsy.2026.10174 41705443 PMC12978994

